# Multiple Active Contours Driven by Particle Swarm Optimization for Cardiac Medical Image Segmentation

**DOI:** 10.1155/2013/132953

**Published:** 2013-05-09

**Authors:** I. Cruz-Aceves, J. G. Aviña-Cervantes, J. M. López-Hernández, S. E. González-Reyna

**Affiliations:** Universidad de Guanajuato, División de Ingenierías, Campus Irapuato-Salamanca, Carretera Salamanca, Valle de Santiago km 3.5+1.8, Comunidad de Palo Blanco, 36885 Salamanca, GTO, Mexico

## Abstract

This paper presents a novel image segmentation method based on multiple active contours driven by particle swarm optimization (MACPSO). The proposed method uses particle swarm optimization over a polar coordinate system to increase the energy-minimizing capability with respect to the traditional active contour model. In the first stage, to evaluate the robustness of the proposed method, a set of synthetic images containing objects with several concavities and Gaussian noise is presented. Subsequently, MACPSO is used to segment the human heart and the human left ventricle from datasets of sequential computed tomography and magnetic resonance images, respectively. Finally, to assess the performance of the medical image segmentations with respect to regions outlined by experts and by the graph cut method objectively and quantifiably, a set of distance and similarity metrics has been adopted. The experimental results demonstrate that MACPSO outperforms the traditional active contour model in terms of segmentation accuracy and stability.

## 1. Introduction

Computed tomography (CT) scanning and magnetic resonance imaging (MRI) are effective and widely used modalities in clinical practice for the diagnosis of cardiac disease. The process carried out by a cardiologist is based on a visual examination of the images followed by a manual delineation of the human organ. This process can be subjective, time-consuming, and susceptible to errors. According to the above process, the accurate medical image segmentation by computational techniques plays an essential role.

Image segmentation is an important and challenging task in computer vision and image processing areas with different applications including medical image analysis, video surveillance, biology, and militar, systems. In recent years, numerous approaches have been introduced for this purpose based only on information available in the image such as wavelet transform [1], rule optimization with region growing [2], enhanced suppressed fuzzy c-means [3], improved watershed transform [4], multithreshold using artificial immune systems [5], graph cut [6, 7], and active contour models (ACM) [8, 9]. This latter technique was introduced by [10], and it is an energy-minimizing spline that consists of control points known as snaxels. The spline will evolve through the evaluation of internal and external forces according to the shape of the object of interest. ACM has been widely used in medical applications including segmentation of breast lesions [11], breast tumors [12], human prostate [13], and intravascular, ultrasound images [14], to name a few.

There are two main drawbacks in the traditional implementation of active contour models. Firstly, initialization of control points must be close to the object of interest; otherwise, failure of convergence will occur. Secondly, the snake is prone to stagnate in local minima and results in an inaccurate convergence to the boundaries of the object. To address these drawbacks, many researchers have suggested to adapt different techniques to work together with the active contour models including waterballoons [15], statistical methods [16, 17], graph cut [18], genetic algorithms [19], differential evolution [20], and particle swarm optimization (PSO) [21] where static large searching windows are dynamically generated depending on the initial position of the interactive control points. Similarly, in [22], a snake model hybrid was proposed by adapting the PSO velocity equation to the active contour model. The performance of both PSO approaches is very suitable according to the tests since the active contour model becomes more robust in local minima problem.

PSO has become very popular to solve optimization problems in continuous spaces [23, 24]. PSO is similar to evolutionary computation techniques since it handles a set of randomly initialized potential solutions known as swarm instead of population. These potential solutions are referred to as particles rather than individuals, and they are evaluated using a fitness function. This computational intelligence technique provides a mechanism inspired by the cognitive and social behavior of bird flocking or fish schooling to exchange information between particles flown through hyperspace based on two main ways generally. Firstly, all the particles are guided by the best particle of the swarm, and, secondly, each particle keeps track of its best solution found through iterations, which is an advantage with regard to some evolutionary computation techniques. As PSO is not computationally expensive and it is highly efficient, it has been used in medical applications such as branch-cut phase unwrapping of MRI data [25] and tumor classification [26].

In this paper, we introduce a new method based on multiple active contours driven by particle swarm optimization (MACPSO) to segment an object of interest by dividing the search space into polar sections. Each polar section has a swarm of particles composed of control points, which performs a strategy search with the aim of finding the optimal control point (snaxel) in its particular constrained space. MACPSO method is able to overcome in a very suitable way the inaccurate convergence to the concave boundaries of the object and the drawback of initialization of the traditional ACM. In addition, the proposed method also addresses the problem of segmenting datasets of sequential CT and MR images which contain the human heart and the human left ventricle, respectively. Finally, to visualize the sequential CT segmentations obtained from MACPSO, a 3D reconstruction approach of the human heart is presented.

The structure of this work is as follows. In Section 2, the basics of active contour model and particle swarm optimization are presented. In Section 3, the proposed MACPSO method is introduced, along with a set of similarity metrics to assess its performance. The experimental results are discussed in Section 4, and from the numerical analysis, conclusions are presented in Section 5.

## 2. Background

In this section, the fundamentals of the active contour model and particle swarm optimization are explained in detail.

### 2.1. Active Contour Model

Active contour model (ACM), also called snake, is a parametric curve that can move within a spatial image domain where it was defined. The snake is described by *p*(*s*, *t*) = (*x*(*s*, *t*),  *y*(*s*, *t*)),  *s* ∈ [0,1], where *t* is the time-related changing aspect. This curve evolves through time to minimize the total energy function given by the following:

(1) $$\begin{array}{c} E_{\text{snake}}=\int_{0}^{1}\left[E_{\mathrm{int}}\left(p\left(s,t\right)\right)+E_{\text{ext}}\left(p\left(s,t\right)\right)\right]\text{d}s. \end{array}$$

The defined energy function consists of two components, *E*_int⁡_ and *E*_ext_ that represent the internal and external energies, respectively. The internal energy presented in the following is used to maintain the search within the spatial image domain and the shape modification of the parametric curve:

(2) $$\begin{array}{c} E_{\mathrm{int}}\left(p\left(s,t\right)\right)=\frac{1}{2}\left[\alpha \left(s\right){\left|\frac{\partial p(s)}{\partial s}\right|}^{2}+\beta \left(s\right){\left|\frac{\partial^{2}p\left(s\right)}{\partial s^{2}}\right|}^{2}\right]. \end{array}$$

Internal energy is represented by the first derivative of *p*(*s*) controlled by curve tension parameter *α*(*s*) and the second derivative of *p*(*s*) guided by rigidity parameter *β*(*s*).

The external energy presented in the following is defined by the particular features of the image, where ∇*I*(*p*(*s*)) is the surface gradient calculated at *p*(*s*) and *γ* is the weight parameter of this force:

(3) $$\begin{array}{c} E_{\text{ext}}\left(p\left(s\right)\right)=-\gamma {\left|\nabla I\left(p\left(s\right)\right)\right|}^{2}. \end{array}$$

The optimal solution is acquired by solving the following Euler equation, that is, when external and internal forces become stable:

(4) $$\begin{array}{c} \nabla E_{\text{ext}}-\alpha \frac{\partial^{2}p\left(s\right)}{\partial s^{2}}+\beta \frac{\partial^{4}p\left(s\right)}{\partial s^{4}}=0. \end{array}$$

The computational implementation of ACM is conformed by a set of *n* discrete points {*p*_*i*_ | *i* = 1,2,…, *n*}. The discrete formulation of internal energy is computed by (5), and the external energy is approximated by (6). In both external and internal energies,  (*q*_*i*,*j*_)  is the control point  (*p*_*i*_), and  (*j*)  is the index in the searching window. In addition, the local energy function given by (7) is iteratively evaluated in order to minimize the *k*_*i*_ index by using (8), where *W*_*i*_ represents the predefined searching window for the control point *p*_*i*_ [21]:

(5) $$\begin{array}{c} E_{\mathrm{int}}=\frac{1}{2}\left[\alpha \left(s\right){\left|q_{i,j}-p_{i-1}\right|}_{2}^{2}+\beta \left(s\right){\left|p_{i-1}-2q_{i,j}+p_{i+1}\right|}_{2}^{2}\right], \end{array}$$

(6) $$\begin{array}{c} E_{\text{ext}}=-\gamma {\left|\nabla I\left(q_{i,j}\right)\right|}_{2}^{2}, \end{array}$$

(7) $$\begin{array}{c} E_{i,j}=E_{\mathrm{int}}+E_{\text{ext}}, \end{array}$$

(8) $$\begin{array}{c} E_{\text{snake}}=\sum_{i=1}^{n}E_{i,k_{i}},\;k_{i}=\arg\;\underset{j}{\min}\left(E_{i,j}\right),\;\;j\in W_{i}. \end{array}$$

The traditional ACM has two main drawbacks: firstly, sensitivity to the initial position of the control points. Secondly, the control points are prone to stagnate in local minima due to the presence of noise in the image deflecting the curve of the optimum edge. A suitable alternative to overcome the local minima drawback is to use a robust optimization technique as particle swarm optimization, which is described in Section 2.2.

### 2.2. Particle Swarm Optimization

PSO is a population-based computational intelligence technique developed by [23, 24] to solve optimization problems. As in evolutionary computation techniques, the population (referred to as swarm in PSO) consists of a number of potential solutions known as individuals (called particles in PSO) to the optimization task. Each particle moves through hyperspace to a new position according to the following velocity equation:

(9) vi(t+1)=φvi(t)+κr1(pbest−xi(t))+κr2(pgbest−xi(t)),

where *v*_*i*_(*t*) is the current velocity of the particle *x*_*i*_,  (*t*) denotes the time step, *φ* is the inertia weight, *κ* represents the learning factor, *r*_1_,  *r*_2_ ~ *U*(0,1) where *U* is a uniform distribution, *p*_best_ is the current best solution found by the present particle, and *p*_*g*best_ is the current best solution found by the best particle of the whole swarm. Assuming that the new velocity of the particle has been updated, its new position is computed by using the following:

(10) $$\begin{array}{c} x_{i}\left(t+1\right)=x_{i}\left(t\right)+v_{i}\left(t+1\right). \end{array}$$

According to the previous description, the PSO algorithm can be implemented by using the following procedure.

1. Set the swarm size and initialize each particle by generating random candidate solutions and velocities.
2. Evaluate each particle in the predefined fitness function and update its *p*_best_ only if the current fitness is better.
3. Find the particle that has the best fitness in the whole swarm and update *p*_*g*best_ only if the fitness value found is better.
4. If the stopping criterion is satisfied (e.g., stability or number of iterations), then stop.
5. Update velocity and position of all the particles according to (9) and (10), then repeat steps (2)–(5).

In Section 3, the proposed image segmentation method is described in detail.

## 3. Proposed Image Segmentation Method

The proposed MACPSO method based on particle swarm optimization and multiple active contours is described in Section 3.1. Additionally, to evaluate the performance of the proposed method, the set of similarity metrics is explained in Section 3.2.

### 3.1. Multiple Active Contours Driven by Particle Swarm Optimization (MACPSO)

**Figure 1:**
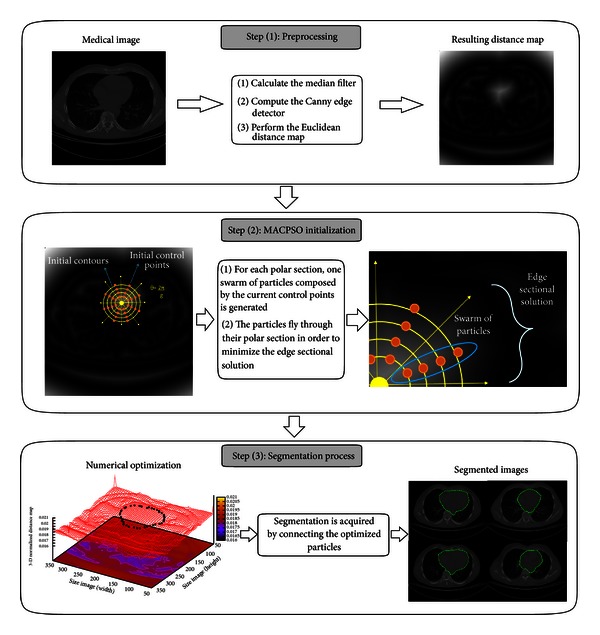
Process of the proposed MACPSO image segmentation method.

Due to the two main drawbacks of the traditional ACM discussed above, PSO is adopted to drive multiple active contours dividing the object of interest into a polar optimization problem. Since the methodology of the proposed MACPSO method makes it possible to apply the traditional implementation of PSO, some advantages are inherently acquired such as robustness, low computational time, and efficiency. The procedure of the proposed segmentation method is illustrated in Figure 1, and it is described below.

In the preprocessing stage of MACPSO, we first remove the noise of the image by utilizing a 2D median filter (3 × 3 window size). Subsequently, the Canny edge detector (*σ* = 1.3, *T*_*l*_ = 10.0, and *T*_*h*_ = 30.0) is used to detect the edge between the background and regions of interest. In the final step of this stage, in order to perform the minimization process, the Euclidean distance map is produced. The second stage is the MACPSO initialization on the resulting distance map, where the origin point of the coordinate system can be determined by the user in an interactive way or it can be generated automatically inside the region of interest. The generated coordinate system divides the region or object of interest through *θ* = 2*π*/*g*, where *g* denotes the degrees of each constrained polar section *S*. On the other hand, the *n* initial contours can be created in a circular or elliptical shape according to the pattern of the region of interest and assuming that this region is within their spatial domain. After the *n* contours are produced, *n* control points (snaxels) are generated and assigned as particles for each constrained polar section *S*_*i*_, in which one edge sectional solution and one swarm of particles *O*_*i*_ must exist. The segmentation process is performed by applying the PSO strategy in each section *S*_*i*_ separately in order to be placed on its corresponding edge sectional solution. For each section, the particles are evaluated according to the fitness function corresponding to external energy derived from (6), and through iterations the best particle (*g*_best_) of each swarm is updated only if a best value is found in its search space. When the optimization process for each swarm is finished, the resulting segmented object is acquired by connecting the *g*_best_ particle of each swarm to each other.

This proposed method has three main advantages in the initialization process. Firstly, the initial contours can be automatically initialized in a circular or elliptical shape. Secondly, the number of snaxels can be adjusted directly by modifying the number of sections in which the object of interest is divided. These two features must be considered to adapt this method according to the shape of the object of interest and obtain a more accurate segmentation without affecting the PSO performance. The third advantage is the origin point created interactively by the user, which is used to generate automatically all the snaxels on the spatial domain of the object of interest. Due to this advantage, the proposed method is easy to extend in the segmentation of sequential CT and MR images by just reproducing the origin point through the set of images.

The procedure of the proposed MACPSO image segmentation method is described as follows.

1. Initialize coordinates (*x*, *y*) from the origin point, degrees *g*, and number of snakes.
2. Initialize parameters of PSO algorithm: number of iterations, inertia weight, and learning factor.
3. Generate one swarm for each polar section *S*_*i*_ assigning the current snaxels as particles.
4. For each swarm *O*_*i*_, initialize velocities and assign the initials  *p*_best_  and *g*_best_.
  - Apply restriction of the search space to ignore improper particles.
  - Evaluate each particle in fitness function.
  - Update *p*_best_ and *g*_best_ if better particles are found.
  - Apply (9) and (10), respectively.
  - If the stopping criterion is satisfied (e.g., stability or number of iterations), then stop; otherwise, go to step (a).
5. Stop MACPSO.

### 3.2. Validation Metrics

To assess the performance of the proposed method in medical image segmentation, the Jaccard index, the Dice index, the Hausdorff distance, and area and perimeter metrics have been adopted to be compared with the traditional ACM and the regions outlined by two experts.

The Jaccard index *J*(*A*, *B*) and the Dice index *D*(*A*, *B*) are similarity measures situated in the range [0,1] used for binary variables [4]. These indexes are calculated by using (11) and (12), respectively. In our tests, *A* represents the segmented region by computational methods (MACPSO and traditional active contour model separately) and *B* represents the region outlined by the experts. In both indexes, if regions *A* and *B* are entirely overlapping, the obtained result is 1, and 0 it is when these two regions are completely different:

(11) $$\begin{array}{c} J\left(A,B\right)=\frac{A\cap B}{A\cup B}, \end{array}$$

(12) $$\begin{array}{c} D\left(A,B\right)=\frac{2\left(A\cap B\right)}{A+B}. \end{array}$$

The Hausdorff distance is a commonly used metric for shape matching in medical image segmentation. It measures the degree of similarity between two superimposed sets and it is calculated by the following, where *a* and *b* are points defined in sets *A* and *B*, respectively, and ||*a* − *b*||  is some underlying distance (Euclidean distance in our tests):

(13) $$\begin{array}{c} H\left(A,B\right)=\underset{a\in A}{\max\;}\underset{b\in B}{\min}||a-b||. \end{array}$$

In Section 4, the segmentation results in different synthetic and medical images using the proposed MACPSO method and being analyzed by the validation metrics are presented.

## 4. Experimental Results

In this section, we evaluate the performance of the proposed MACPSO method for segmenting objects on different medical and synthetic images. The computational implementations presented in this section are performed using the gcc compiler version 4.4.5 running on Debian GNU/Linux 6.0, Intel Core i3 with 2.13 Ghz and 4 Gb of memory.

### 4.1. Application on Synthetic Images

**Figure 2:**
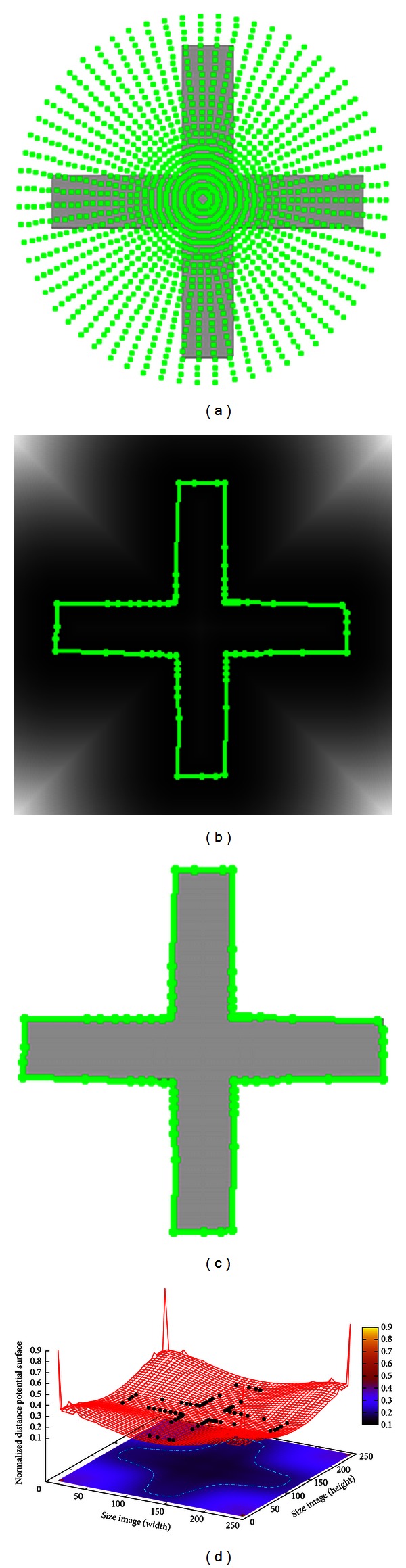
Symmetrical cross: (a) initialization of the proposed method, (b) result of MACPSO on the Euclidean distance map derived from cross-object, (c) result of MACPSO implementation, and (d) result of optimization process on the distance potential surface.

**Figure 3:**
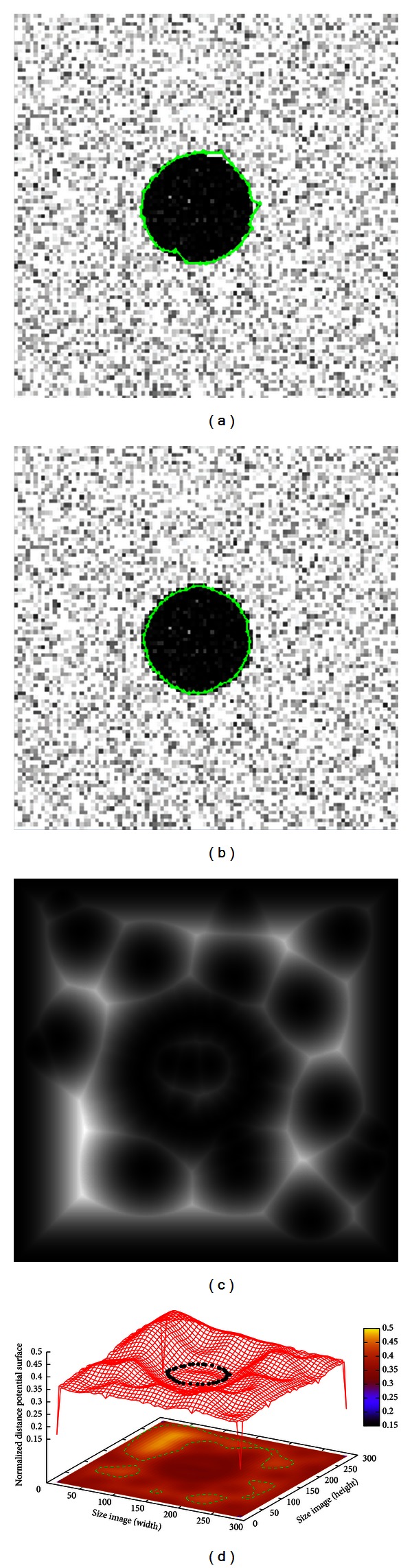
Noisy circle: (a) result of traditional ACM, (b) result of MACPSO implementation, (c) Euclidean distance map of the image, and (d) result of optimization process on the distance potential surface.

**Figure 4:**
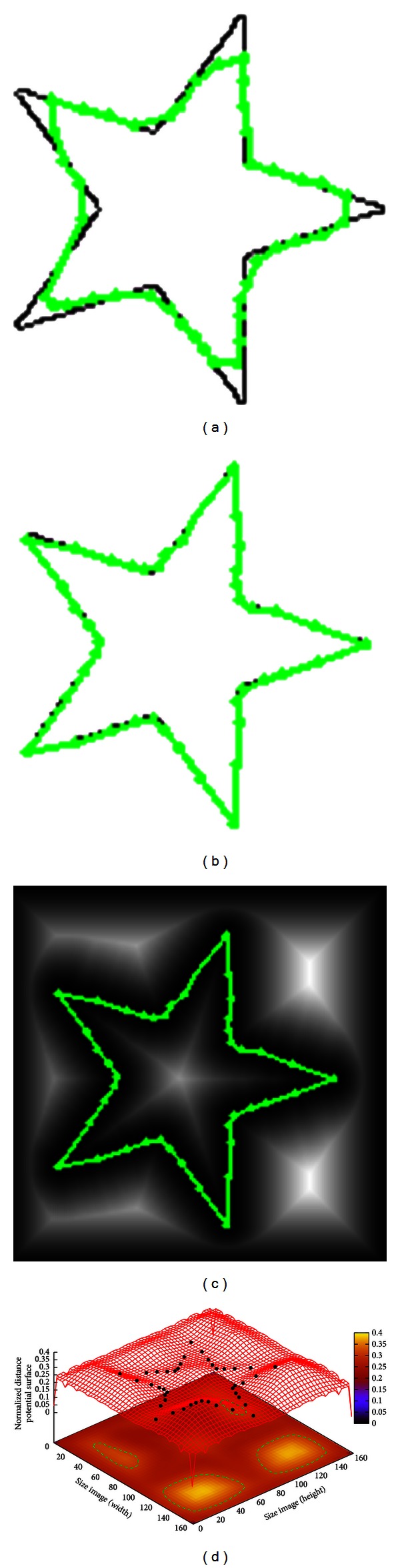
Synthetic star: (a) result of traditional ACM, (b) result of MACPSO implementation, (c) result of MACPSO on the Euclidean distance map, and (d) result of optimization process on the distance potential surface.

The results of segmenting synthetic images are shown in Figures 2, 3, and 4, which are a symmetrical cross, circle with Gaussian, noise and a star object, respectively. The three synthetic images have been used to test the performance of other approaches such as [21, 22] which are described below.

In Figure 2, the result of applying MACPSO on a symmetrical cross-image of size 256 × 256 pixels is presented. In Figure 2(a), the initialization of the proposed method is shown. This simulation is performed by using the next parameters: 20 iterations, 0.8 of inertia weight, 0.5 of learning factor, 25 snakes, and 63 control points per snake obtained since the value of *g* is 10. Additionally, after the preprocessing stage, the Euclidean distance map (EDM) is derived from the image to perform the segmentation process. In Figure 2(b), the result of applying MACPSO on the EDM is presented, which, in Figure 2(c), is shown on the original test image. This segmentation process must be viewed as an optimization task since the snaxels (particles) work on the 3D distance map also called distance potential surface computed from the EDM in order to minimize the shape of the object of interest. The resulting particles on the distance potential surface when the optimization process is finished are shown in Figure 2(d) where these particles are subsequently connected to obtain the final segmentation result previously introduced in Figure 2(c). In this test image, the MACPSO method can overcome the concavity problem and converge into a correct way to the cross-edge on the image in 0.277 s. 

Figure 3 presents a synthetic image of size 300 × 300 pixels containing a circle with Gaussian noise (mean = 0 and variance = 0.04). As shown in Figure 3(a), the result of applying the traditional implementation of active contour model cannot overcome the Gaussian noise to fit the object boundary accurately. The curve tension *α*, rigidity *β*, and weight external energy *γ* parameters remain constant according to experiments performed by [21], where similar segmentation problems have been effectively addressed. The traditional AC parameters in this simulation are set as *α* = 0.01, *β* = 0.9, *γ* = 0.05, and 42 control points giving an executing time of 0.104 s. Moreover, as shown in Figure 3(b), the proposed method is robust in the presence of noise and it is able to locate the circle boundary in an accurate way. The inertia, learning factor, and iteration parameters of MACPSO are statistically adjusted to promote local exploitation, while the number of snakes has been considered to enclose the object of interest, and number of snaxels to smooth and fit the resulting contour. In this simulation, MACPSO parameters are set as iterations = 20, inertia weight = 0.8, learning factor = 0.5, number of snakes = 15, and 42 snaxels per snake since *g*-value = 15 with an executing time of 0.159 s. In Figure 3(c), the Euclidean distance map after the preprocessing stage is presented and the distance potential surface with the resulting optimized control points is shown in Figure 3(d).

In Figure 4, a synthetic image of size 160 × 160 pixels with an artificial shape of a star is introduced.  Figure 4(a) presents the resulting segmentation obtained with the traditional implementation of ACM using the parameters as *α* = 0.01, *β* = 0.9, *γ* = 0.05, and 42 control points giving an executing time of 0.090 s. In this figure the traditional ACM is not able to fit the concavities of the object boundary accurately. This drawback is solved with the proposed method by overcoming the concavity problem and fitting the correct boundary as shown in Figure 4(b). In this simulation, the MACPSO parameters are set as iterations = 20, inertia weight = 0.8, learning factor = 0.5, number of snakes = 15 and 42 snaxels per snake since the value of *g* is equal to 15 with an executing time of 0.125 s. On the other hand, derived from the star object, in Figures 4(c) and 4(d), the resulting segmentation on the Euclidean distance map and the convergence of the optimized particles on the distance potential surface are illustrated.

The quality of the segmented objects through MACPSO in the three different synthetic test images has demonstrated that the proposed method is more stable and accurate than the traditional implementation of ACM. The robustness of MACPSO is due to the process of convergence carried out by particle swarm optimization instead of the traditional ACM. Even though the computational time of the proposed method is comparable to the traditional ACM, MACPSO capabilities, such as avoiding local minima and fitting to the boundary of the objects, improve the quality of the obtained segmentations.

In Section 4.2, based on the performance of MACPSO on synthetic images, a set of cardiac medical images has been introduced to prove the accuracy of the proposed method through different distance and similarity measures.

### 4.2. Application on Medical Images

The proposed method has been applied in the segmentation of datasets from sequential CT and MR images which contain the human heart and the human left ventricle, respectively. These medical images have been provided by the Mexican Social Security Institute and by the Auckland MRI Research Group, University of Auckland.

**Figure 5:**
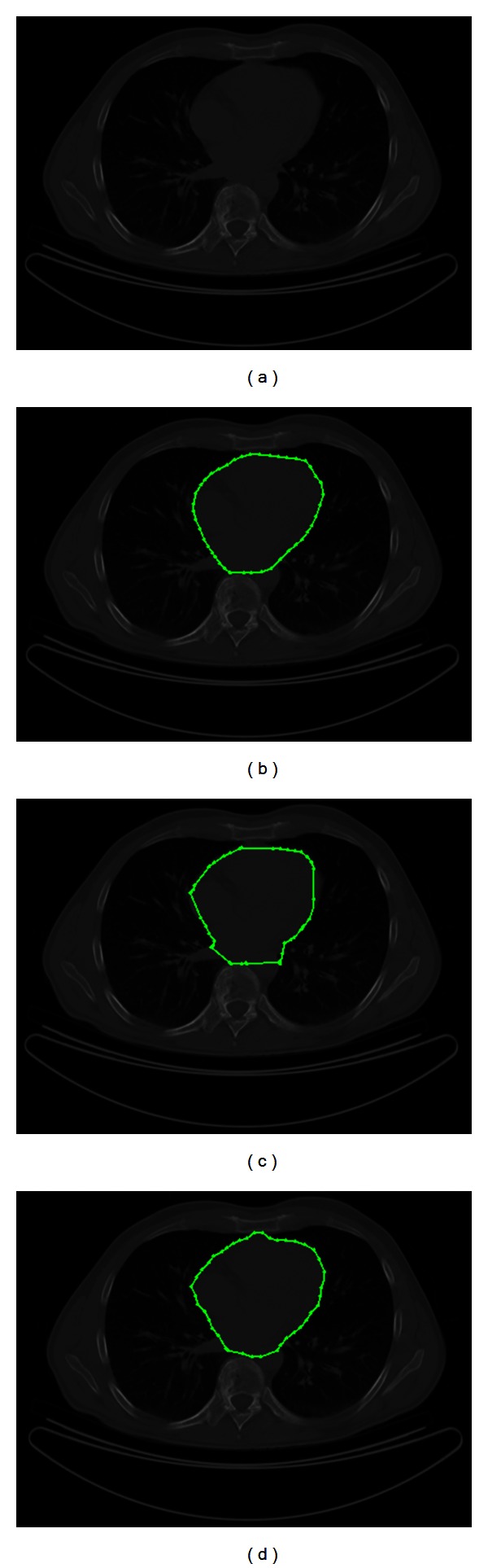
CT image: (a) test image, (b) the human heart outlined by experts, (c) result of traditional ACM and (d) result of MACPSO implementation.

Figure 5(a) shows a medical image of size 512 × 512 pixels acquired from a CT scanning with the aim of segmenting the present human heart. In Figure 5(b), the human heart outlined by cardiologists is presented. In addition, Figure 5(c) illustrates the segmented region through the traditional implementation of ACM with the next parameters: 42 control points, *α* = 0.01, *β* = 0.9, and *γ* = 0.05 in 0.087 s. As shown in Figure 5(d), the human heart segmentation by using the proposed MACPSO method fits the heart boundary accurately. The parameters in this simulation are set as iterations = 20, inertia weight = 0.8, learning factor = 0.5, number of snakes = 15, and *g*-value = 15 (obtaining 42 snaxels per snake) with an executing time of 0.127 s. 

**Figure 6:**
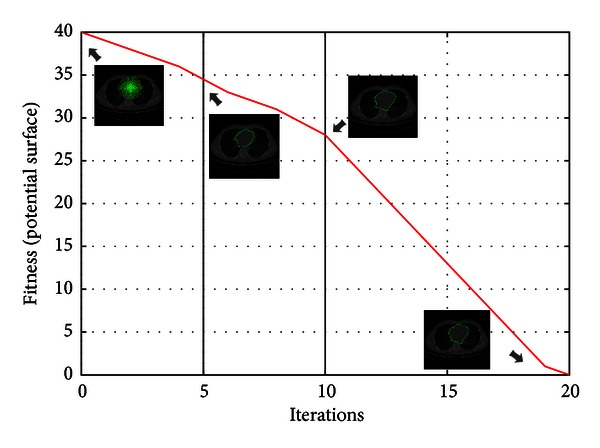
Convergence of the human heart segmentation through PSO iterations in CT image.

Figure 6 shows the process of convergence of the MACPSO on CT test image. The convergence measure is given by the fitness value which is computed from the average of the control points on the distance potential surface and it is iteratively minimized through the 20 predefined iterations to improve the human heart segmentation. 

**Figure 7:**
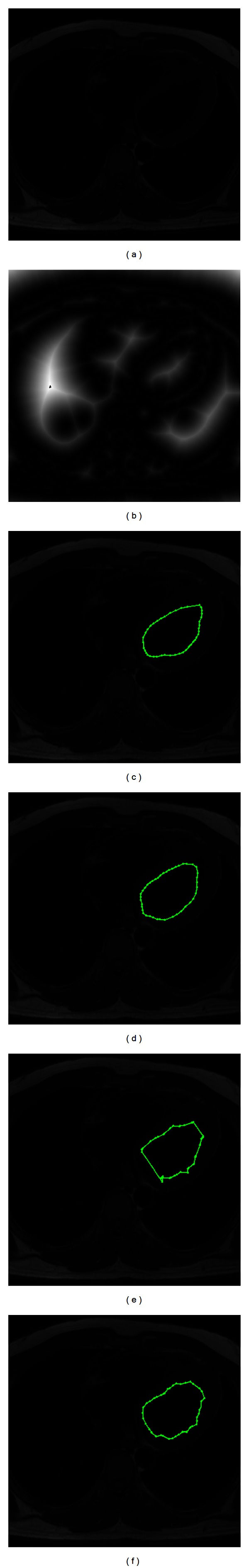
MR image: (a) test image, (b) Euclidean distance map of test image, (c) the human left ventricle outlined by expert 1, (d) the human left ventricle outlined by expert 2, (e) result of traditional ACM, and (f) result of MACPSO implementation.

Figure 7(a) shows a low-contrast, 512 × 512 pixels medical image acquired from the MR procedure with the aim of segmenting the present human left ventricle. In Figure 7(b), the Euclidean distance map derived from the test image is presented to increase the perception of the segmentation task. On the other hand, in Figures 7(c) and 7(d), the human left ventricle outlined by expert 1 and expert 2 is presented.  Figure 7(e) shows the resulting segmented region through the traditional implementation of ACM with the next parameters: 42 control points, *α* = 0.01, *β* = 0.9, and *γ* = 0.05 in 0.085s.  Figure 7(f) illustrates the resulting segmentation by using the proposed MACPSO method locating the boundary human left ventricle accurately. The parameters in this simulation are set as iterations = 20, inertia weight = 0.8, learning factor = 0.5, number of snakes = 9, and *g*-value = 15 (obtaining 42 snaxels per snake) with an executing time of 0.108 s.

**Figure 8:**
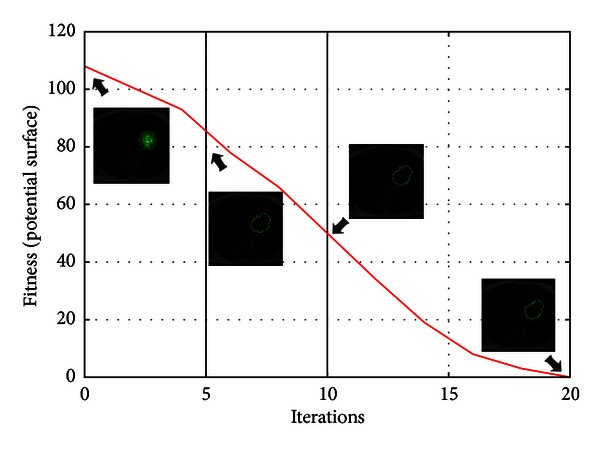
Convergence of the human left ventricle segmentation through PSO iterations in MR image.

Figure 8 illustrates the process of convergence of the MACPSO on an MR test image by using the average of the control points as a fitness value evaluated on the distance potential surface on each iteration. This convergence is performed with the same parameters used in Figure 7. 

Due to the initialization methodology of MACPSO, this method can be easily extended to work with datasets of sequential images just reproducing the coordinates (*x*, *y*) of the origin point and the initial parameters in the whole set of images. This initialization process is an advantage over the traditional implementation of ACM, since only one user interaction is needed to generate automatically all of snaxels to the segmentation process, while in traditional ACM each snaxel has to be provided interactively, which is time consuming.

**Figure 9:**
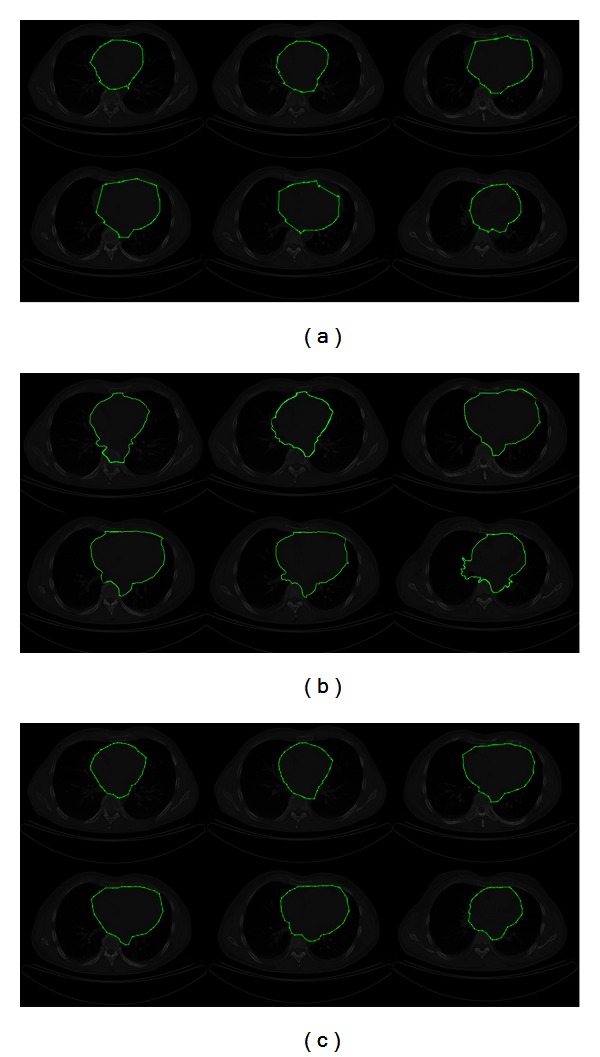
CT images (human heart segmentation): (a) results of traditional ACM, (b) results of graph cut method, and (c) results of MACPSO implementation.

In Figure 9 the results of segmenting a subset of CT images containing the human heart are presented. These images have been extracted from a segmented dataset consisting of 144 sequential CT images from different patients where each image is of size 512 × 512 pixels. In Figure 9(a), the resulting segmentations of applying the traditional ACM are presented, in which the fitting problem is shown. The parameters of ACM are set as 42 control points, *α* = 0.01, *β* = 0.9, and *γ* = 0.05 with an executing time of 9.168 s.  Figure 9(b) presents the segmentation results obtained through the interactive graph cut method, which were computed in 10.065 s. In this method, the experts defined areas (human heart and background seeds) that should be separated by the segmentation. Moreover, in Figure 9(c), the segmented images by using MACPSO show in a very suitable way the boundary of the human heart. In this simulation, the parameters of the proposed method are set as iterations = 20, inertia weight = 0.8, learning factor = 0.5, number of snakes = 15, and *g*-value = 15 (obtaining 42 snaxels per snake) with an executing time of 11.152 s.

From the previously dataset of CT images described above, in Table 1 the average of the resulting segmentations performed by two experts, traditional ACM, graph cut, and the MACPSO method is listed. The comparative results suggest that the MACPSO method is promising in human heart segmentation.

**Figure 10:**
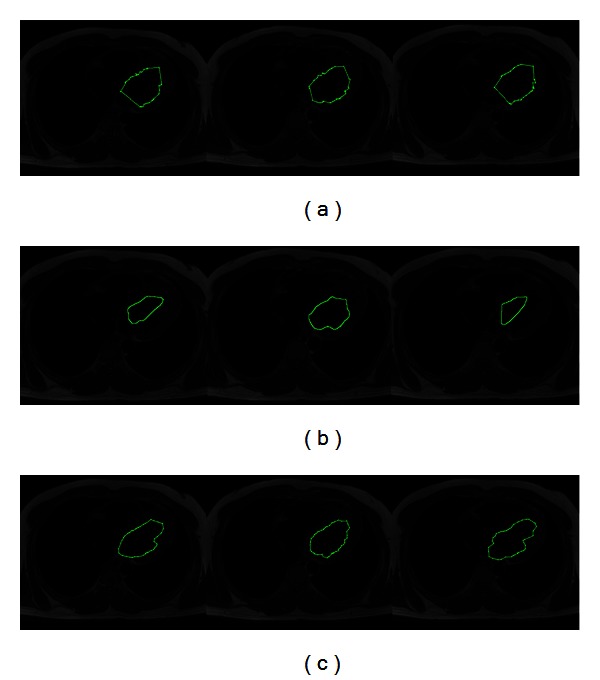
MR images (human left ventricle segmentation): (a) results of traditional ACM, (b) results of graph cut method, and (c) results of MACPSO implementation.

In Figure 10, the results of segmenting a subset of MR images containing the human left ventricle are presented. These images have been extracted from a segmented dataset with 23 sequential MR images of a patient where each image is of size 512 × 512 pixels.  Figure 10(a) shows the resulting segmentations of applying the traditional ACM, where the resulting snake cannot adjust to the correct left ventricle boundary. The parameters of ACM are set as 42 control points, *α* = 0.01, *β* = 0.9, and *γ* = 0.05 with an executing time of 4.183 s. To perform the graph cut method, the experts defined the human left ventricle and background seeds. In Figure 10(b), the segmentation results acquired by the interactive graph cut method, which were obtained in 3.726 s, are illustrated. On the other hand, in Figure 10(c), the segmented images via MACPSO show in an appropriate way the boundary of the human left ventricle. In this simulation, the parameters of the proposed method are set as iterations = 20, inertia weight = 0.8, learning factor = 0.5, number of snakes = 9, and *g*-value = 15 (obtaining 42 snaxels per snake) with an executing time of 5.179 s.

Moreover, to quantify the resulting segmentations from the dataset of sequential MR images described above, Table 2 presents comparative results through the Hausdorff distance, the Jaccard index, and the Dice index. This similarity analysis shows that the proposed method is very suitable in left ventricle segmentation. Additionally, area and perimeter measures have been adopted to compare in a quantitative way the segmented regions performed by two experts, traditional ACM, graph cut, and MACPSO method, which are shown in Table 3.

As shown in Table 1, compared to segmentations made by experts, the distance and similarity measures indicate that MACPSO is promising in human heart segmentation on CT images since the Jaccard and Dice indexes show a high accuracy (90% and 95% with expert 2) and the Hausdorff distance is low with respect to the traditional ACM and graph cut method. Besides, Table 2 shows that the performance of MACPSO in human left ventricle is more sensitive due to the low contrast and the presence of noise in MR images achieving an acceptable accuracy of 83%. The area and perimeter measures have also shown that MACPSO is more stable than ACM and graph cut, since the values of the proposed method are located in the range of the values acquired by the experts.

**Figure 11:**
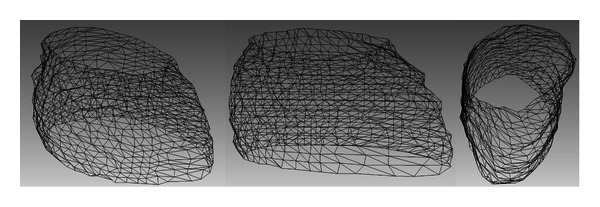
3D reconstruction of human heart from the segmented cardiac CT images.

Since MACPSO has proved a suitable efficiency in the segmentation of sequential cardiac images, a potential application of the proposed method is the 3D reconstruction of human organs. The quality of the reconstruction depends on the number of sequential images considered. In Figure 11, a 3D reconstruction approach of the human heart is presented, which is achieved by superimposing the resulting contours according to the image acquisition order, and triangulation is performed through the snaxels of each contour to obtain a complete mapping. This 3D reconstruction consists of 18 CT images previously selected by the experts and segmented via the proposed MACPSO method.

## 5. Conclusions

In this research, a new image segmentation method based on multiple active contours driven by particle swarm optimization (MACPSO) has been presented. MACPSO divides the search space in polar sections to overcome the sensitivity to initial contour position and the local minima drawbacks of the traditional active contour model (ACM). In a first stage of this paper, to evaluate the performance of the proposed method and to compare it to the traditional ACM, some experiments with synthetic images have been introduced. Subsequently, in the second stage, experiments with cardiac medical images acquired from the computed tomography and magnetic resonance procedures have been used. The experimental results revealed that the proposed method can lead to more efficiency and stability in the presence of noise and concavities than the traditional ACM. This advantage made it possible to obtain a high accuracy and effectiveness in the human heart and human left ventricle segmentations compared to those outlined by the experts and by the graph cut method according to the evidence of similarity metrics. Additionally, the experimental results have also shown that the local exploitation of polar sections through the constant parameters of MACPSO is highly suitable for medical image applications, including segmenting datasets of sequential medical images within an appropriate computational time.

## Figures and Tables

**Table 1:** Average similarity measure with the Jaccard index, the Dice index, and the Hausdorff distance among the regions segmented by the traditional ACM, graph cut method, our proposed method (MACPSO), and the regions outlined by two experts of the set of CT images.

Comparative studies	Distance/similarity measure		
	Hausdorff (*H*)	Jaccard's index (*J*)	Dice's index (*D*)
ACM versus Expert 1	7.071	0.5272	0.6904
ACM versus Expert 2	5.0	0.5	0.6666
Graph cut versus Expert 1	4.2426	0.7142	0.8333
Graph cut versus Expert 2	3.1622	0.6153	0.7619
MACPSO versus Expert 1	2.0	0.8260	0.9047
MACPSO versus Expert 2	1.4142	0.9090	0.9523

**Table 2:** Average similarity measure with the Jaccard index, the Dice index, and the Hausdorff distance among the regions segmented by the traditional ACM, graph cut method, our proposed method (MACPSO), and the regions outlined by two experts of the set of MR images.

Comparative studies	Distance/similarity measure		
	Hausdorff (*H*)	Jaccard's index (*J*)	Dice's index (*D*)
ACM versus Expert 1	7.615	0.377	0.5476
ACM versus Expert 2	15.231	0.4	0.5714
Graph cut versus Expert 1	6.236	0.5555	0.7142
Graph cut versus Expert 2	6.782	0.5272	0.6904
MACPSO versus Expert 1	6.708	0.7142	0.8333
MACPSO versus Expert 2	7.071	0.6153	0.7619

**Table 3:** Average of the area and perimeter in pixels obtained from the traditional ACM, graph cut method, our proposed method (MACPSO), and the regions outlined by two experts from the sets of CT and MR images.

Method	Set of CT images		Set of MR images	
	Area	Perimeter	Area	Perimeter
Expert 1	9904.5 pix.	355.209 pix.	5796.0 pix.	291.645 pix.
Expert 2	10369.5 pix.	370.137 pix.	6250.5 pix.	310.13 pix.
ACM	9529.5 pix.	367.634 pix.	7283.5 pix.	350.734 pix.
Graph cut	10036.5 pix.	410.673 pix.	7405.0 pix.	383.992 pix.
MACPSO	10439.5 pix.	376.902 pix.	6385.5 pix.	308.009 pix.
